# Management of Primary and Secondary Hyperparathyroidism in a Patient With Chronic Kidney Disease (CKD)

**DOI:** 10.7759/cureus.94038

**Published:** 2025-10-07

**Authors:** Bashirat O Johnson, Sunggeun Lee

**Affiliations:** 1 Internal Medicine, New York Medical College, Metropolitan Hospital Center, New York City, USA; 2 Nephrology, New York Medical College, Metropolitan Hospital Center, New York City, USA

**Keywords:** calcitriol, calcium monitoring, cinacalcet, hyperparathyroidism treatment, kdigo, kidney disease: improving global outcomes (kdigo) classification, parathyroidectomy

## Abstract

An 82-year-old female with stage 4 chronic kidney disease (CKD) presented with asymptomatic hypercalcemia and elevated parathyroid hormone (PTH) levels.

Workup confirmed primary hyperparathyroidism due to a parathyroid adenoma, with secondary hyperparathyroidism due to CKD. Given her age and surgical risk, she was managed medically with cinacalcet, later switched to calcitriol due to hypocalcemia, then reverted to cinacalcet after recurrent hypercalcemia.

This case illustrates the diagnostic and therapeutic challenges of managing coexisting primary and secondary hyperparathyroidism in CKD, highlighting the importance of individualized, guideline-based medical therapy and close biochemical monitoring.

## Introduction

Primary hyperparathyroidism is a well-documented clinical condition, characterized by asymptomatic hypercalcemia and elevated levels of parathyroid hormone (PTH), with the most common etiology being parathyroid adenoma [1].

Secondary hyperparathyroidism is the most common cause of elevated PTH in patients with chronic kidney disease (CKD) [2]. The major biochemical difference between these two types of hyperparathyroidism is the low serum calcium levels in secondary hyperparathyroidism versus the high calcium levels in primary hyperparathyroidism, with both having elevated PTH levels.

Here we present the dilemma of managing a complex case of confirmed primary hyperparathyroidism in a patient diagnosed with CKD stage 4, who also exhibited features of secondary hyperparathyroidism, with further decline in renal function.

## Case presentation

An 82-year-old Hispanic female with a history of hypertension and stage 4 CKD was referred for nephrology evaluation. Her chronic medications included nifedipine XL 30 mg once daily, carvedilol 3.125 mg twice daily, and furosemide 40 mg once daily.

Initial laboratory workup revealed a serum creatinine of 2.1 mg/dL, with an estimated glomerular filtration rate (eGFR) of 23 mL/min/1.73 m³. Serum calcium was 10.5 mg/dL (albumin: 4.1 g/dL), indicating hypercalcemia; phosphorus was within normal limits at 3.3 mg/dL. Serum 25-hydroxyvitamin D was normal (38.2 nmol/L), and PTH was markedly elevated at 549 pg/mL. The differential diagnoses included primary hyperparathyroidism, secondary hyperparathyroidism related to CKD, multiple myeloma, and familial hypocalciuric hypercalcemia (FHH). A 24-hour urinary calcium excretion after discontinuing furosemide was low (<1 mg/dL), raising the suspicion of FHH. However, genetic testing for FHH (testing for CASR, AP2S1, and GNA11 genes) was negative, excluding the diagnosis.

**Figure 1:**
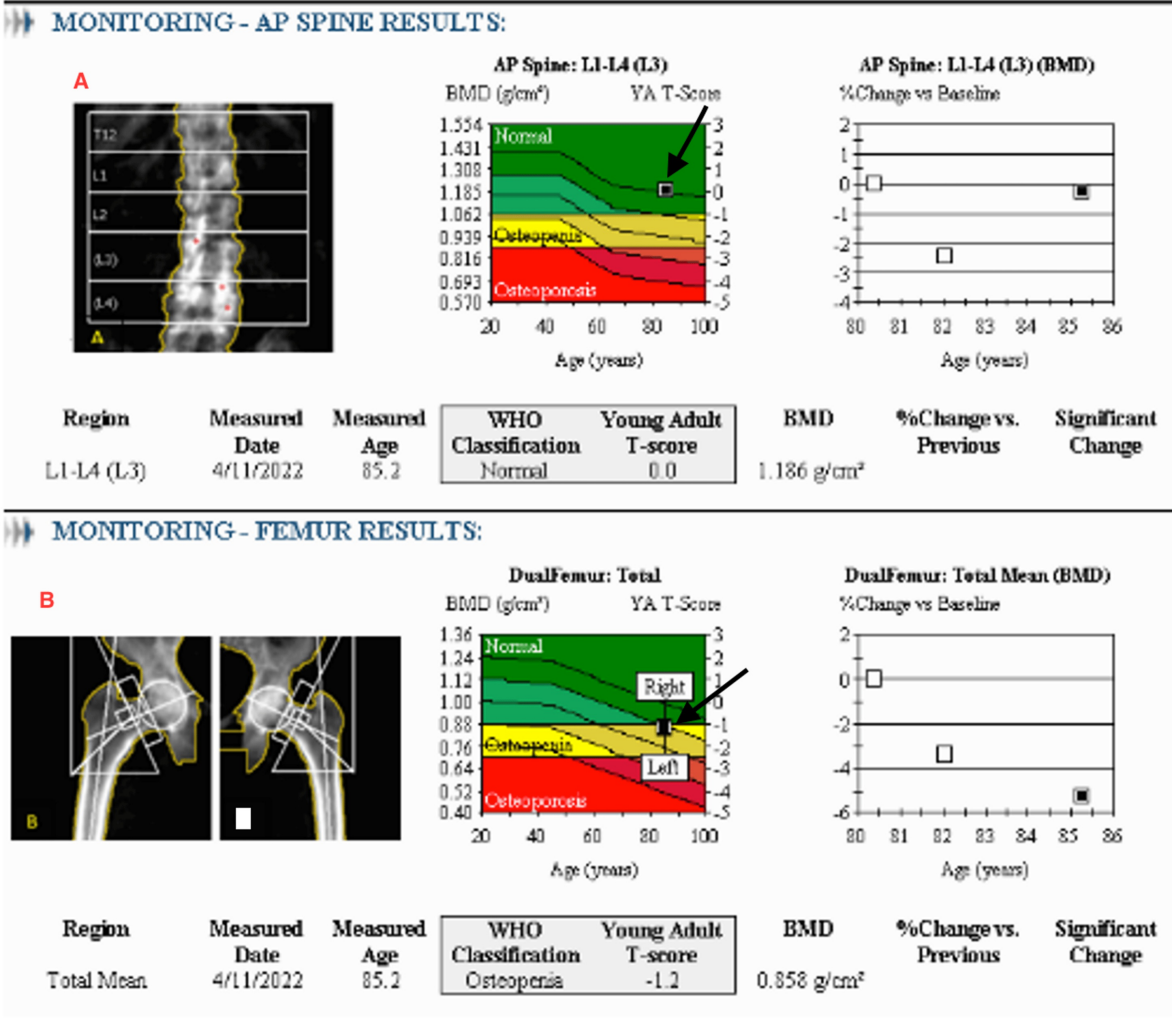
Dual-energy X-ray absorptiometry (DEXA) scan report Dual-energy X-ray absorptiometry (DEXA) scan demonstrating normal bone mineral density (BMD) in the anteroposterior (AP) view of the lumbar spine (Panel A), with a BMD of 1.186 g/cm² and a corresponding T-score of 0 (see arrow), indicative of normal bone health. Panel B reveals evidence of osteopenia of both the right and left femur, with a BMD of 0.858 g/cm² and a T-score of -1.2 (see arrow), consistent with low bone mass.

Further evaluation with abdominal ultrasonography and non-contrast CT imaging showed no evidence of nephrolithiasis. A dual-energy X-ray absorptiometry (DEXA) scan revealed osteopenia of both femurs (Figure 1).

Serum protein electrophoresis was negative for monoclonal proteins. Kappa and lambda free light chains were within normal limits, with a kappa/lambda free light chain ratio of 1.44 (normal). Urine protein electrophoresis and immunofixation showed no monoclonal bands, effectively ruling out a monoclonal gammopathy.

**Figure 2:**
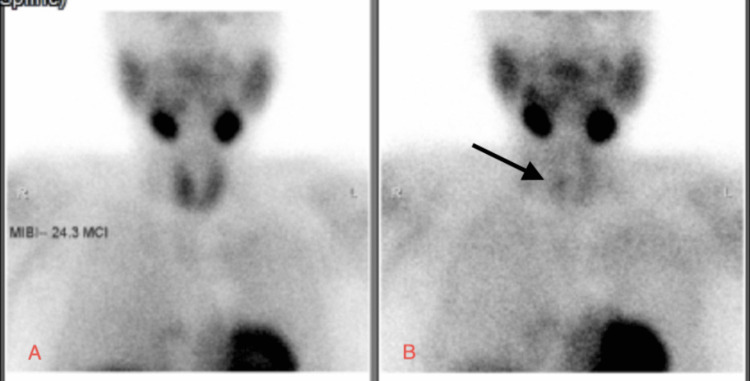
Early (A) and delayed (B) planar images from parathyroid scintigraphy demonstrating a small solitary focus of radiotracer uptake on delayed imaging (arrow), located at the mid-pole region of the right lobe of the thyroid gland, suggestive of a parathyroid adenoma.

Parathyroid scintigraphy demonstrated a parathyroid adenoma (Figure 2), confirming a diagnosis of primary hyperparathyroidism.

However, given the patient’s advanced age, asymptomatic status, absence of nephrolithiasis, and limited surgical candidacy, endocrine evaluation recommended conservative management, without parathyroidectomy.

Medical therapy with cinacalcet, a calcimimetic agent, was initiated at 30 mg daily and titrated to 30 mg twice daily. Over the course of five years, serum calcium levels gradually declined, eventually stabilizing before reaching a low point of 7.1 mg/dL, with the development of hyperphosphatemia (phosphorus of 5.9 mg/dL). PTH levels initially decreased to 256 pg/ml, remained stable for a while, and gradually increased to a high of 1205 pg/ml (Table 1), indicating the onset of secondary hyperparathyroidism.

**Table 1:** Serum calcium and intact PTH levels in response to cinacalcet and calcitriol Ref.: reference range; PTH: parathyroid hormone

Stage of treatment	Calcium level (Ref. 8.6 - 10.2 mg/dL)	Phosphorus (Ref. 2.5 - 4.5 mg/dL)	Intact PTH (pg/mL)
At presentation (baseline)	10.5	3.3	549
After five years on cinacalcet	7.1	5.9	1205
After two years on calcitriol	10.7	3.8	670
Median levels after stopping calcitriol and restarting cinacalcet	9.6	5.1	416

As a result, cinacalcet was discontinued, and oral calcitriol, an active vitamin D analog, was initiated at 0.25 mcg daily, along with a non-calcium-based phosphate binder. However, subsequent monitoring revealed recurrence of hypercalcemia (peak 10.7 mg/dL), a known side effect of calcitriol therapy, leading to its discontinuation and re-initiation of cinacalcet. A progressive decline in renal function was, however, observed over the years, ultimately requiring initiation of dialysis within seven years.

With ongoing follow-up, serum calcium levels were successfully maintained within the target range (9.0 - 10.1 mg/dL), and PTH levels remained stable between 290 and 570 pg/mL (Table 1). 

This treatment sequence underscores the challenges of maintaining a balance between calcium and PTH levels, requiring ongoing adjustments in response to biochemical changes, allowing for effective management of both primary and secondary hyperparathyroidism, with treatment decisions guided by serial monitoring and alignment with Kidney Disease: Improving Global Outcomes (KDIGO) recommendations [3].

## Discussion

The most common etiology of hypercalcemia in patients with elevated PTH levels is primary hyperparathyroidism, usually due to a parathyroid adenoma. FHH, a close differential, was also considered in this patient, especially given the low urinary calcium excretion. However, it is important to note that urinary calcium levels can be misleading in the context of advanced CKD, where calcium metabolism is significantly altered. This occurs as a result of low production of active vitamin D from the kidneys, leading to low calcium absorption in the gut and increased tubular calcium reabsorption in an attempt to compensate for the low gut absorption, ultimately resulting in decreased calcium excretion [2, 4]. This phenomenon is seen in patients with secondary hyperparathyroidism, suggesting that our patient had both secondary and primary hyperparathyroidism when first assessed. In addition, the calcium-to-creatinine ratio, a commonly used tool for distinguishing FHH from primary hyperparathyroidism, could not be accurately calculated due to the patient's impaired kidney function. 

Genetic testing for known FHH-associated mutations was negative, making the likelihood of FHH very low. However, due to the possibility of undetected genetic variants or somatic mosaicism, particularly in older adults, FHH could not be completely ruled out. 

This patient was diagnosed with a parathyroid adenoma, as suggested by the parathyroid scan, which was significant enough to cause hypercalcemia.

With progression of CKD, particularly by stage 3a, CKD-mineral bone disease (MBD) often develops, characterized by biochemical abnormalities in calcium, phosphorus, and PTH, as well as vascular and valvular abnormalities [3]. 

Initially, she presented with asymptomatic hypercalcemia and elevated PTH levels, indicating primary hyperparathyroidism. However, at that time, there was likely a contributing component of secondary hyperparathyroidism, evident from the low urinary calcium and severely elevated PTH levels, which could not be explained by FHH.

This case is unique because the patient initially presented with features of primary hyperparathyroidism. If left untreated, this condition can result in end-organ damage due to persistently elevated calcium levels. Overactivity of the parathyroid gland leads to complications such as kidney stones, osteoporosis, cognitive decline, and poorly controlled hypertension, as well as vascular and valvular calcifications [3]. She was not considered a surgical candidate due to her advanced age and the infeasibility of the procedure. She was also stable and asymptomatic, with no evidence of nephrolithiasis.

Studies have shown that asymptomatic patients over 50 years of age, with stable serum calcium levels less than 1 mg/dl above the upper limit of normal, no evidence of osteoporosis or vertebral fractures, no kidney stones, and low urinary calcium levels can be safely monitored for up to 10 years without surgery [5,6].

Although the patient had pre-existing CKD, which preceded the onset of the hypercalcemia, she did not meet the criteria for parathyroidectomy [6]. Patients in this group should be followed up for worsening of the hypercalcemia, hypercalciuria, or new nephrolithiasis over a period of 10 years [5].

Several studies indicate that hypercalcemia does not worsen significantly in asymptomatic patients who are followed up for up to three years [7, 8]. However, in certain cases, hypercalcemia may worsen, necessitating parathyroidectomy. This was demonstrated in a study of 191 patients with asymptomatic primary hyperparathyroidism, followed over two years, where 8% of the study population required parathyroidectomy [7] due to significant worsening of hypercalcemia. Other studies following patients for a longer duration, up to 15 years, have reported similar findings, with most patients maintaining stable biochemical parameters [9, 10].

This patient was commenced on oral cinacalcet, a calcimimetic agent that activates the calcium-sensing receptor in the parathyroid gland to inhibit secretion of PTH [11]. 

Cinacalcet has been shown to be the only calcimimetic available for the management of hypercalcemia in both primary and secondary hyperparathyroidism, particularly in CKD, reducing the need for parathyroidectomy [12, 13]. In addition to lowering PTH levels, cinacalcet also reduces serum calcium and phosphorus levels [3]. While cinacalcet is generally well tolerated, randomized trials have demonstrated its efficacy in patients with asymptomatic hypercalcemia, although it does not provide a biochemical cure when compared with parathyroidectomy [14]. Surgery, on the other hand, when indicated, normalizes PTH levels and reverses the end-organ effects of primary hyperparathyroidism while improving quality of life [8, 14].

In patients with both primary and secondary hyperparathyroidism undergoing treatment with calcimimetics, regular monitoring of PTH, calcium, and phosphorus levels is essential [3]. A common side effect of cinacalcet is hypocalcemia, which may be severe enough to require switching the patient to a vitamin D analog, provided the serum PTH exceeds twice the upper limit of the reference range [3,11]. However, if PTH is below this threshold, a vitamin D analog will not be initiated, as further PTH suppression can lead to hungry bone syndrome [11]. In such cases, calcium-based phosphorus binders may be used if phosphorus levels are elevated, with continued monitoring of calcium, phosphorus, and PTH levels, as well as monitoring for vascular and valvular calcifications [3].

Following a reduction of serum calcium levels by cinacalcet, secondary hyperparathyroidism in our patient became more apparent, as her corrected serum calcium levels dropped below the reference range, with high phosphorus and persistently elevated PTH levels. At this point, the decision was made to switch to a synthetic, active vitamin D analog (calcitriol).

Hyperparathyroidism in CKD is associated with a faster decline in renal function, as PTH is a uremic toxin and is an independent risk factor for cardiovascular events and death [15, 16]. Vitamin D supplementation is the first-line treatment in patients with non-dialysis CKD. Several guidelines recommend testing for 25-hydroxyvitamin D levels first, as low vitamin D is associated with high PTH levels [16]. If vitamin D deficiency is found, supplementation with vitamin D (such as cholecalciferol) is advised to suppress PTH, increase serum calcium levels, and maintain the normal calcium-phosphate homeostasis through a tightly controlled feedback cycle [3, 16].

In patients with CKD stages 4-5, with severe and progressive hyperparathyroidism, calcitriol is recommended because the kidneys are unable to convert 25-hydroxyvitamin D to the active 1,25-dihydroxyvitamin D due to reduced activity of 1-alpha hydroxylase in the kidney [16]. Studies show that active vitamin D is associated with a slower progression to end-stage renal disease and improved cardiovascular outcomes [17, 18]. In advanced CKD with hypocalcemia, calcitriol helps to raise the serum calcium while monitoring calcium and phosphorus levels.

Although hypercalcemia and hyperphosphatemia are the most common side effects of systemic administration of calcitriol, it is generally well tolerated, with other side effects like hypercalciuria and nephrolithiasis occurring in less than 10% of cases [19]. With this in mind, close monitoring of calcium and phosphorus levels is required, with the initiation of phosphorus binders if indicated and consideration of reintroducing cinacalcet if calcium levels become elevated in the setting of high PTH levels [20].

The recommended starting dose of calcitriol is 0.25 mcg daily, with a gradual dose increase to 0.5 mcg daily based on the patient's response, while carefully monitoring calcium and phosphorus levels and maintaining PTH levels within the recommended range [3].

In this patient, initiation of calcitriol resulted in progressive elevation of calcium levels and subsequent hypercalcemia, necessitating a transition back to cinacalcet. Since then, serum calcium has been consistently maintained within the normal range through regular monitoring. Additionally, there has been a significant reduction in PTH levels, which have been sustained at two to nine times the upper limit of normal, in accordance with KDIGO guidelines [3].

## Conclusions

This complex case underscores the diagnostic and therapeutic challenges involved in managing hypercalcemia in a patient with both primary and secondary hyperparathyroidism. While initial findings pointed towards primary hyperparathyroidism caused by parathyroid adenoma, the overlapping features of secondary hyperparathyroidism complicated the clinical picture, especially with the progression of CKD. Given that the patient was not a candidate for surgery, medical management became the primary approach. Treatment was initiated with cinacalcet, the medication of choice for patients with hypercalcemia and CKD. As treatment progressed, the patient developed hypocalcemia, a common side effect of calcimimetics, along with persistently elevated PTH levels, prompting discontinuation of cinacalcet and introduction of calcitriol, an active vitamin D analog, to help restore calcium balance and suppress PTH secretion. Continuous biochemical monitoring remained essential throughout treatment, as vitamin D analogs carry the risk of inducing hypercalcemia, which may require a return to cinacalcet therapy.

This case highlights the importance of personalized treatment approaches, ongoing laboratory monitoring, and flexibility in adjusting therapy in response to changes in mineral metabolism, particularly in the setting of CKD-MBD.
